# Multi-function PtCo nanozymes/CdS nanocrystals@graphene oxide luminophores and K_2_S_2_O_8_/H_2_O_2_ coreactants-based dual amplified electrochemiluminescence immunosensor for ultrasensitive detection of anti-myeloperoxidase antibody

**DOI:** 10.1186/s12951-021-00968-4

**Published:** 2021-07-29

**Authors:** Wei Yang, Zheng Zhou, Haiping Wu, Changjin Liu, Bo Shen, Shijia Ding, Yonglie Zhou

**Affiliations:** 1 Department of Clinical Laboratory, Zhejiang Provincial People’s Hospital, People’s Hospital of Hangzhou Medical College, Hangzhou, 310014 China; 2 Department of Clinical Laboratory, Chongqing University Three Gorges Hospital, Chongqing, 404000 China; 3 Key Laboratory of Clinical Laboratory Diagnostics (Ministry of Education), College of Laboratory Medicine, Chongqing Medical University, Chongqing, 400016 China

**Keywords:** PtCo/CdS@GO luminophores, Nanozyme, K_2_S_2_O_8_/H_2_O_2_ coreactants, Electrochemiluminescence immunosensor, Anti-myeloperoxidase antibody

## Abstract

**Background:**

Anti-myeloperoxidase antibody (anti-MPO) is an important biomarker for anti-neutrophil cytoplasm antibody (ANCA)-associated vasculitides (AAVs). However, the complicated operation procedures and insufficient sensitivity of conventional anti-MPO detection methods limit their application in monitoring efficacy of AAVs in clinical diagnosis. Herein, a dual amplified electrochemiluminescence (ECL) immunosensor based on multi-function PtCo nanozymes/CdS nanocrystals@graphene oxide (PtCo/CdS@GO) luminophores and K_2_S_2_O_8_/H_2_O_2_ coreactants has been fabricated for ultrasensitive detection of anti-MPO.

**Results:**

PtCo/CdS@GO luminophores as novel signal amplification labels and nanocarriers to load rabbit anti-mouse IgG were synthesized by co-doping with Pt and Co nanozymes simultaneously with several considerable advantages, including astonishing peroxidase-like catalytic activity, high-efficiency luminescence performance and superior stability in aqueous solutions. Meanwhile, upon the K_2_S_2_O_8_/H_2_O_2_ coreactants system, benefiting from the efficient peroxidase-like activity of the PtCo/CdS@GO toward H_2_O_2_, massive of transient reactive intermediates could react with K_2_S_2_O_8_, thus obtaining higher ECL emission. Therefore, the developed ECL immunosensor for anti-MPO detection displayed good analytical performance with good concentration linearity in the range of 0.02 to 1000 pg/mL and low detection limit down to 7.39 fg/mL.

**Conclusions:**

The introduction of multi-function PtCo/CdS@GO luminophores into the established ECL immunoassay not only was successfully applied for specific detection of anti-MPO in clinical serum samples, but also provided a completely new concept to design other high-performance luminophores. Meaningfully, the ECL immunoassay strategy held wide potential for biomarkers detection in clinical diagnosis.

**Graphic abstract:**

**Figure:**
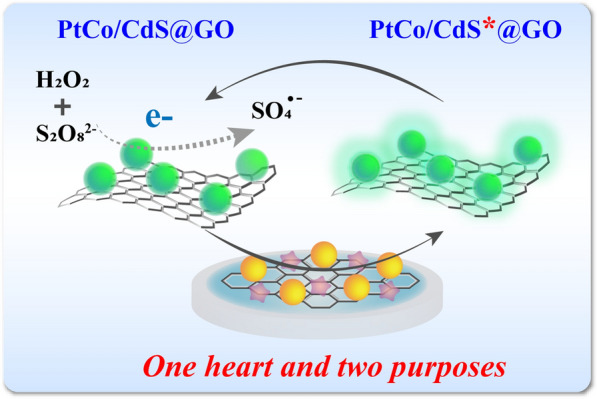

**Supplementary Information:**

The online version contains supplementary material available at 10.1186/s12951-021-00968-4.

## Background

Anti-myeloperoxidase antibody (anti-MPO) is one of the most important circulating biomarker for clinical diagnosis and efficacy monitoring of anti-neutrophil cytoplasm antibody (ANCA)-associated vasculitides (AAVs) [1, 2]. According to the *International Consensus on ANCA Testing in Eosinophilic Granulomatosis with Polyangiitis* and the *Revised 2017 international consensus on testing of ANCAs in granulomatosis with polyangiitis and microscopic polyangiitis*, indirect immunofluorescence (IIF) and enzyme-linked immunosorbent assays (ELISA) have been used to initially screen and verify anti-MPO [3, 4]. However, these conventional anti-MPO detection methods usually suffer from complicated operation procedures, insufficient sensitivity and specificity [5, 6]. Recently, electrochemiluminescence (ECL) immunoassay with the unique advantages of chemiluminescence and electrochemistry, such as low background signal, simplified optical setup, excellent spatial and temporal controllability, has been widely used in the fields of clinical diagnosis, which possesses great promise as a powerful analytical tool to replace IIF and ELISA [7, 8].

In ECL immunoassay system, traditional molecular luminophores (Ru(bpy)_3_^2+^, luminol, acridinium ester) usually have some drawbacks including reagent waste and poor stability, restricting their analytical applications [9, 10]. Thus, with the development of multidisciplinary studies, especially nanomaterial technologies, the emergence of various new types of luminophores have been developed to overcome the aforementioned shortcomings [11–13]. As the potential ECL luminophore candidates, cadmium sulfide nanocrystals (CdS NCs) possess impressively splendid features including narrow and direct band-gap (2.3 eV), excellent electronic/optical properties, size/surface-trap controlled luminescence and good photostability [14, 15]. Nevertheless, despite the great progress made by CdS NCs, their application in ECL immunoassay still face some challenges such as limited luminescence effectiveness and poor stability. Therefore, to address these challenges simultaneously, the exploration of novel CdS NCs is highly desirable in the field of ECL luminophores.

Currently, a variety of excellent designs of CdS NCs have been sequentially developed to enhance the luminescence efficiency, including metal deposition or doping [16, 17], band-gap engineering by forming core/shell structure [18] and coupling with carbon materials [19]. Among various metals dopants, noble metals-based dopants, especially doping platinum (Pt) with CdS NCs not only evidently enhance the ECL emission through perturbing the host energy level or offering a new electron energy level [20], but also act as peroxidase-like nanozymes to generate transient reactive intermediates like free radicals, and react with another substrate quickly, thus further improving the luminescence performance of CdS NCs [21–23]. Furthermore, the peroxidase-like activities can be reconstructed by charge, coating, doping, loading and external fields [24]. For instance, Fang’s group constructed Pt-CdS/g‑C_3_N_4_-MnO_x_ composite photocatalyst for efficient visible-light-driven overall water splitting [25]. However, insufficient stability, low economy and specificity of Pt-based nanozyme still obstacle its broader application in ECL immunoassay system [26]. In response to above-mentioned drawbacks, the combination of Pt-based CdS NCs with another transition metals (Co, Pd, Mo, Ni, etc.) can possess better stability, higher luminescence efficiency and catalytic activity [27, 28], which hold great promise as efficient ECL luminophores to replace conventional luminescent reagents.

Additionally, maintaining high-efficiency luminescence performance of luminophores while having good stability in the most desirable environment, i.e., aqueous solutions is also an extremely important concerns, which is closely related to the dispersion of CdS NCs, and few technologies can meet all the requirements. Recently, two-dimensional (2D) graphene oxide (GO) has been generally utilized as preeminent support matrixes for nanoparticles anchoring and ensuring dispersion [29]. Moreover, the presence of effective electron-hole and abundant functional groups (–OH, –COOH, etc.) on the surface of GO sheets possess enhanced charge transfer, good biocompatibility and stability [30]. In our previous work, PtCo@rGO hybrids with almost no agglomeration has been successfully synthesized, contributing to the higher dispersion and catalytic activity [6]. Inspired by the above research, simultaneously co-doping with PtCo nanozymes, GO-supported PtCo/CdS (PtCo/CdS@GO) luminophores have been in situ synthesized successfully, loading number of PtCo/CdS NCs and ensuring the dispersion to enhance the luminescence efficiency and stability in aqueous solutions. Furthermore, the prepared multi-function PtCo/CdS@GO luminophores could also be employed as efficient peroxidase-like nanozymes to accelerate electrochemical redox processes of coreactants, thus further improving the luminescence performance.

**Scheme 1:**
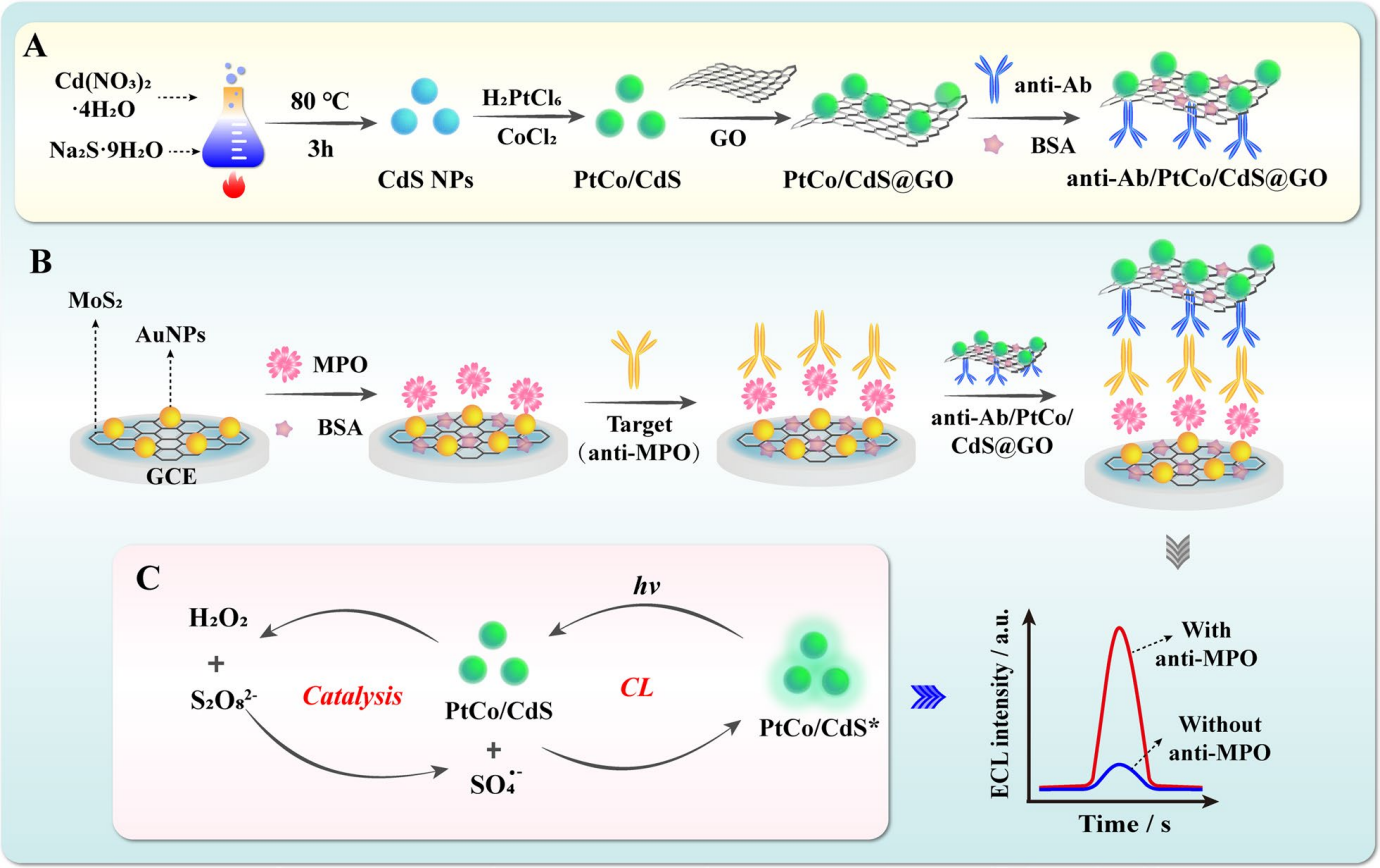
Principle of the ECL immunosensor for anti-MPO detection. **A** The preparation route of the anti-Ab/PtCo/CdS@GO. **B** The construction and detection process of the sandwich-type ECL immunosensor. **C** The amplification mechanism of the PtCo/CdS@GO luminophores

Herein, a dual amplified ECL immunosensor based on multi-function PtCo/CdS@GO luminophores is fabricated for ultrasensitive detection of anti-MPO. As shown in Scheme 1 A, PtCo/CdS NCs are stepwise in situ synthesized by co-doping with Pt and Co dual nanozymes simultaneously and further immobilized on the surface of GO, forming the multi-function PtCo/CdS@GO luminophores. Then, rabbit anti-mouse IgG (anti-Ab) is assembled onto the surface of PtCo/CdS@GO luminophores to fabricate anti-Ab/PtCo/CdS@GO bioconjugates. After that the ECL immunosensor has been constructed by immobilizing MPO (antigen) on the glassy carbon electrodes (GCE) modified with the pre-synthesized Au@MoS_2_ nanosheets, which can greatly improve the electroconductivity and modifiability of the GCE. In the presence of anti-MPO (target), the anti-Ab/PtCo/CdS@GO bioconjugates are assembled on the surface of modified GCE based on sandwich-like immunoreaction. Upon the K_2_S_2_O_8_/H_2_O_2_ coreactants system, benefiting from the strong peroxidase-like activity of PtCo/CdS@GO luminophores toward H_2_O_2_, massive of transient reactive intermediates could react with K_2_S_2_O_8_ to obtain higher ECL emission of PtCo/CdS@GO luminophores, thus further improving the analytical performance of the developed ECL immunosensor. More importantly, the introduction of multi-function PtCo/CdS@GO luminophores into the ECL immunosensor offer an efficient approach for the ultrasensitive detection of anti-MPO and held potential applications in clinical analysis, drug delivery systems and treatment of diseases.

## Materials and methods

### Materials and reagents

Human myeloperoxidase (MPO), rabbit anti-mouse IgG (anti-Ab) and mouse anti-human MPO monoclonal antibody (anti-MPO, target) were purchased from Abcam PLC (Cambridge, UK). Insulin (INS), carcinoembryonic antigen (CEA), α-1-fetoprotein (AFP), prostate specific antigen (PSA), chloroauric acid (HAuCl_4_·3H_2_O), graphene oxide (GO), molybdenum sulfide powder (MoS_2_), chloroplatinic acid (H_2_PtCl_6_), cobalt chloride (CoCl_2_), sodium sulfide (Na_2_S·9H_2_O), cadmium nitrate tetrahydrate (Cd(NO_3_)_2_·4H_2_O), glycine, sodium citrate, bovine serum albumin (BSA, purity ≥ 98 %), glutaraldehyde, polyvinyl pyrrolidone (PVP, MW: 30,000), hydrogen peroxide (H_2_O_2_) and potassium peroxydisulfate (K_2_S_2_O_8_) were obtained from Sigma-Aldrich (St. Louis, USA). All other reagents were of analytical grade, blocking buffer (BSA, 1.0 wt%) and phosphate buffered saline (PBS, 0.01 M, pH 7.4, 2.7 mM KCl, 137 mM NaCl, 1.8 mM KH_2_PO_4_, 8 mM Na_2_HPO_4_) were used Millipore-Q water (≥ 18 MΩ, Milli-Q, Millipore, Germany).

### Instrument

The electrochemiluminescence (ECL) measurements were executed using a MPI-E ECL workstation (Xi’an, China) with a conventional three-electrode system composed of Ag/AgCl electrode as reference, Pt wire as auxiliary and Au@MoS_2_ nanosheets modified glassy carbon electrode (GCE, 3 mm in diameter) as working electrode. Cyclic voltammetry (CV) and electrochemical impedance spectroscopy (EIS) were performed with a PC-controlled CHI 660E electrochemical workstation (Shanghai, China). Transmission electron microscope (TEM, Hitachi, Tokyo, Japan), high-resolution transmission electron microscopy (HRTEM, Talos F200X, USA), scanning electron microscope (SEM, Hitachi SU8000, Japan), energy dispersive spectrometer (EDS, Oxford X-MaxN, Britain) and UV-2550 spectrophotometer (Shimadzu, Kyoto, Japan) were employed to characterize the features and morphologies of nanomaterials.

### Synthesis of PtCo/CdS@GO luminophores

Firstly, 2 mL H_2_PtCl_6_ solution (20 mM), 7 mL CoCl_2_ solution (1.66 mM) and 76 mg glycine were mixed together and stirred for 1 h, and sonicated into an ultrasonic bath at room temperature (RT) for 0.5 h. After that, the well-distributed solution was transferred to a 50 mL Teflon-lined stainless-steel autoclave and heated at 200 °C for 9 h. After being cooled to RT and ultrasonically dispersed for 15 min, 31.0 mg Cd(NO_3_)_2_·4H_2_O was dissolved into the above mixtures (PtCo NCs) and preheated to 80 °C under stirring. Then, 99.4 mg Na_2_S·9H_2_O was dissolved into 5 mL DI water and slowly injected into the above solution. After that, the mixture was heated to 80 °C for 3 h with continuous refluxing. Finally, the obtained products were washed with a mixture buffer (ethanol/acetone, volume ratio = 2/1) for three times (8000 rpm, 15 min) to fully eliminate the excess reactants, and then vacuum-dried at RT for further use. Further, 20 mg PtCo/CdS NCs and 0.2 mL GO (1 mg mL^− 1^) were dispersed in 10 mL of DI water with the assistance of ultrasound, and then gently stirred at RT for 6 h. Finally, the obtained multi-function PtCo/CdS@GO luminophores were centrifuged and washed with ethanol/acetone mixture for 3 times, and then ultrasonically dispersed to a final dispersion concentration of 10 mg mL^− 1^ in 2 mL DI water and stored at 4 °C for further use.

### Synthesis of anti-Ab/PtCo/CdS@GO bioconjugates

100 µL multi-function PtCo/CdS@GO luminophores dispersion (3.33 mg mL^− 1^), 200 µL rabbit anti-mouse IgG (anti-Ab, 1 mg mL^− 1^) and 200 µL PBS (0.01 M, pH 7.4) buffer were mixed together and shaken at 4 °C for 12 h. After that, the as-prepared anti-Ab/PtCo/CdS@GO composites were centrifuged at 7000 rpm for 15 min, and then, 200 µL blocking buffer (BSA, 1.0 wt%) was mixed into the above solution and gently stirred at RT for 1 h to prevent nonspecific binding. Finally, the anti-Ab/PtCo/CdS@GO bioconjugates were collected and washed with PBS buffer for 3 times, and then ultrasonically dispersed in 400 µL PBS (0.01 M, pH 7.4) at 4 °C.

### ECL detection of anti-MPO

The Au nanoparticles functionalized MoS_2_ (Au@MoS_2_) nanosheets were prepared according to our previous work [6], and the modification of the GCE surface was detailed in supplementary materials. Different concentrations of anti-MPO solutions (target, standard solutions or serum samples) were dropped onto the surface of BSA/MPO/Au@MoS_2_/GCE and incubated with at RT for 1 h. Subsequently, as-fabricated anti-Ab/PtCo/CdS@GO bioconjugates (10 µL) was further incubated onto the surface of MPO/BSA/MPO/Au@MoS_2_/GCE at 37 °C for 1 h. After each step, the modified GCE was thoroughly washed with PBS (0.01 M, pH 7.4) buffer to remove unbound substance. Finally, the ECL immunoassay was performed in 4 mL 0.01 M air-saturated PBS (0.01 M, pH 7.4) buffer containing 3 mM H_2_O_2_ and 0.1 M K_2_S_2_O_8_. The potential scanning was ranged from − 1.6 to − 0.6 V, scan rate of 0.1 V s^− 1^ and photomultiplier tube voltage of 800 V.

## Results and discussion

### Characterization of the multi-function PtCo/CdS@GO luminophores

**Fig. 1:**
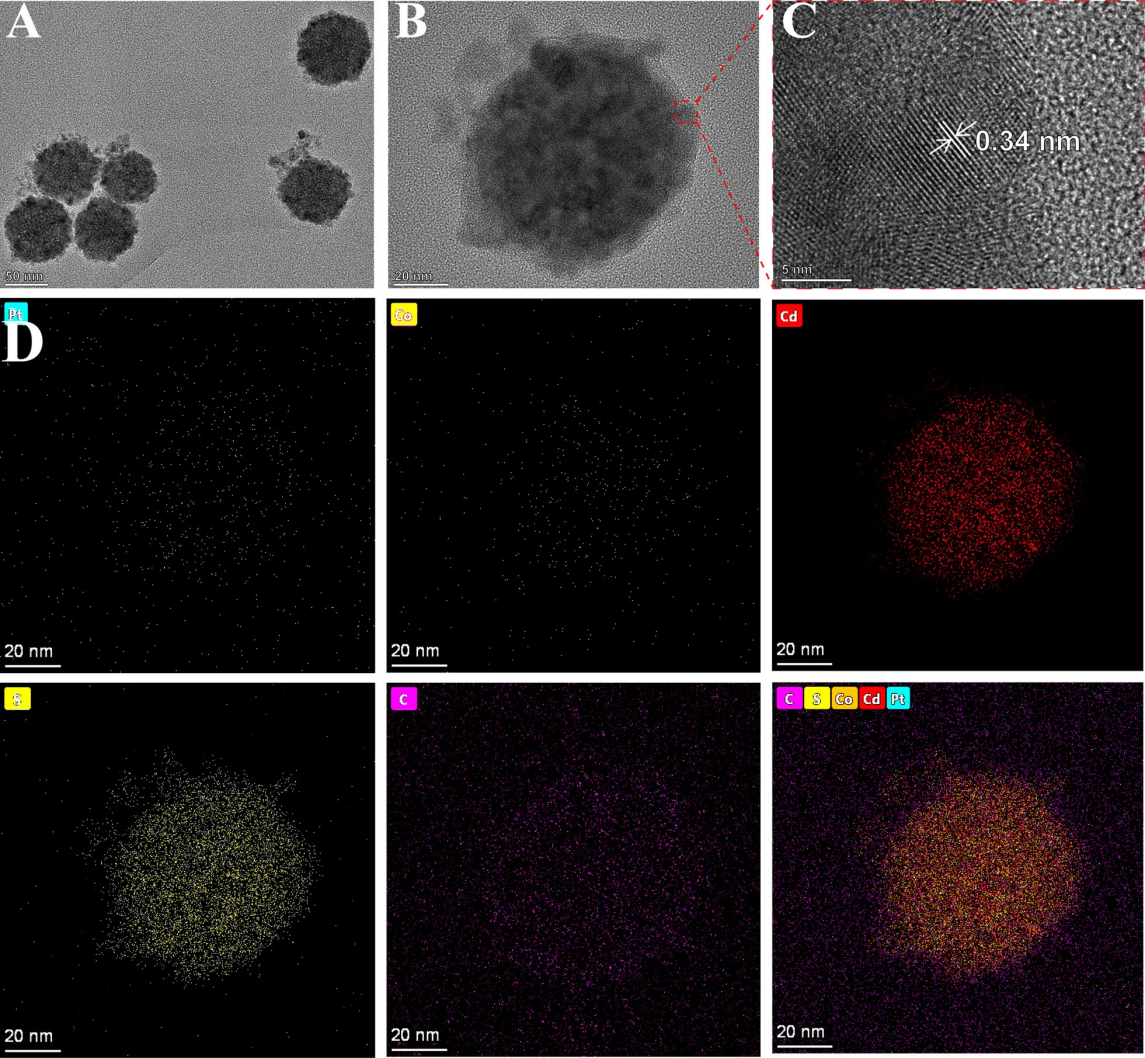
Structural and compositional analyses of the PtCo/CdS@GO luminophores. **A** Low-magnification TEM image of PtCo/CdS@GO luminophores. Scale bar is 50 nm. **B** High-magnification TEM image of PtCo/CdS@GO luminophores. Scale bar is 20 nm; **C** HRTEM image and lattice fringes of PtCo/CdS@GO luminophores. Scale bar is 5 nm. **D** STEM-EDS elemental mapping of PtCo/CdS@GO luminophores. Scale bar is 20 nm

HRTEM, TEM and STEM-EDS elemental mapping have been employed to characterize the size, morphology and elemental composition of the as-prepared nanomaterials, respectively. As displayed in Fig. 1A, B, uniform morphology and well-dispersed PtCo/CdS NCs with an apex-to-apex diameter of about 65–85 nm were distributed on the surface of GO with good stability (Additional file 1: Figure S1). Additionally, STEM-EDS elemental mapping analysis was performed to further confirm the successful synthesis of PtCo/CdS@GO nanocomposites. As shown in Fig. 1D, the Pt, Co, Cd and S element were uniformly distributed onto the entire nanocrystals structure. Moreover, EDS elemental analysis was also used to demonstrate the chemical compositions of PtCo/CdS@GO luminophores. As shown in Additional file 1: Figure S2, the element peaks of Pt (0.55 wt %) and Co (0.15 wt %) were obtained in the EDS image, indicating that the multi-function PtCo/CdS@GO Luminophores were synthesized successfully.

### Characterization of Au@MoS_2_ nanosheets

**Fig. 2:**
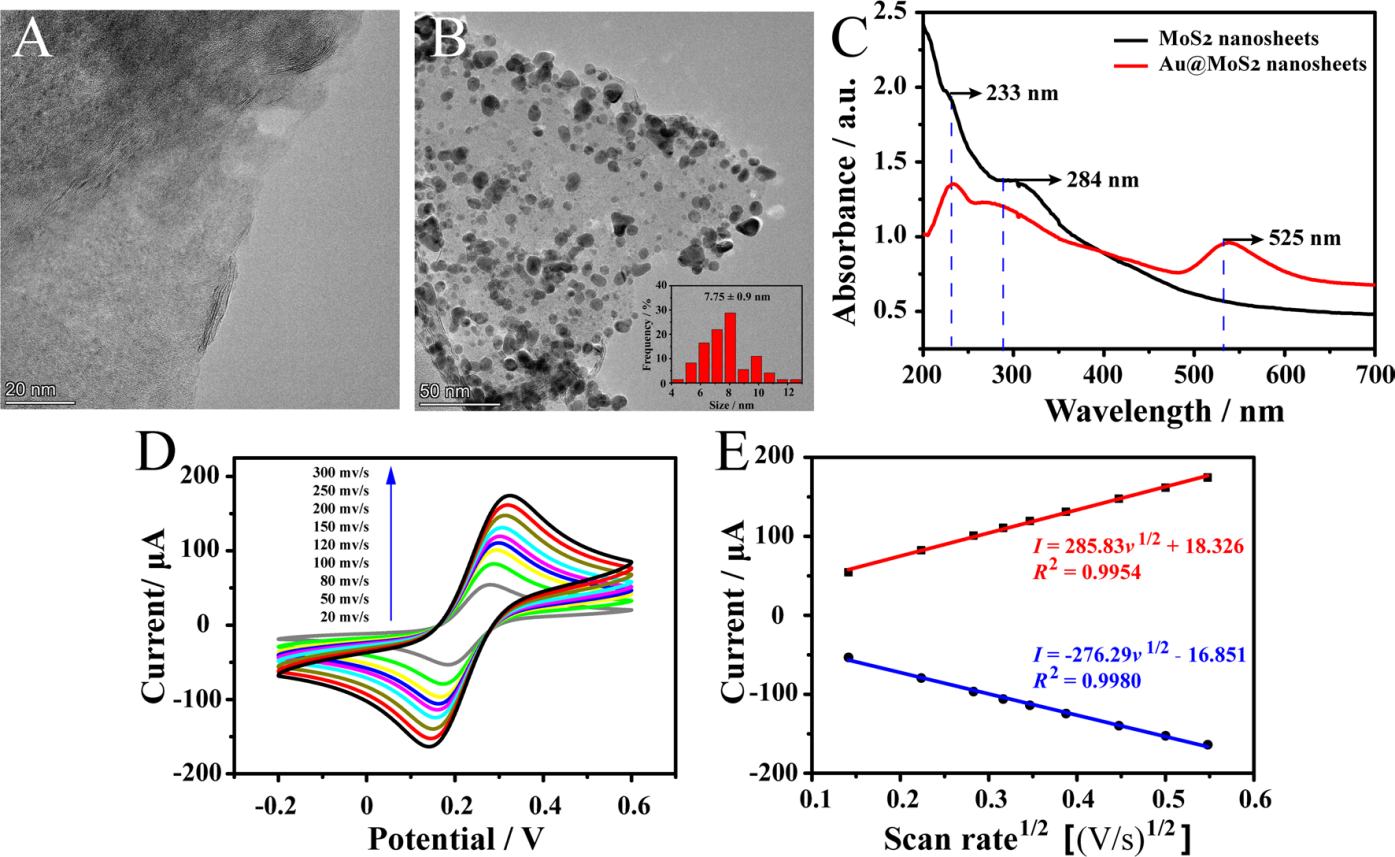
**A** TEM image of MoS_2_. Scale bar is 20 nm. **B** TEM image of Au@MoS_2_ nanosheets and size-distribution histogram of Au NPs (inset). Scale bar is 50 nm. **C** Typical UV-vis absorption spectra of MoS_2_ and Au@MoS_2_ nanosheets. **D** CVs of Au@MoS_2_ modified GCE in reaction buffer with 5 mM Fe(CN)_6_^3−/4−^ and 0.1 M KCl at different scan rates from 20 to 300 mV/s. **E** The linear relations of Au@MoS_2_ modified GCE with the anodic and cathodic peak current against the square root of scan rate

MoS_2_ nanosheets is a kind of layered nanomaterials that can stabilize metal NPs and expand their functions due to its high surface area and two-dimensional ultrathin atomic layer structure [31]. When Au NPs were in situ synthesized on MoS_2_ nanosheets, it could greatly improve the conductivity and modifiability of MoS_2_. Thus, in order to characterize the as-prepared as-synthesized MoS_2_ and Au@MoS_2_ nanosheets, TEM was employed. As TEM results shown in Fig. 2 A, MoS_2_ nanosheets displayed a typical layer-like nanostructure. After in situ reducing with Au NPs, it was clear that Au NPs with the average diameter of 7.75 ± 0.9 nm (Fig. 2B, inset) were homogeneously distributed on the MoS_2_ nanosheets surface. Moreover, for further proving the successful preparation of Au@MoS_2_ nanosheets, UV-vis absorption spectra characterization was employed. As shown in Fig. 2C, MoS_2_ nanosheets exhibited two characteristic absorption peaks at wavelengths about 233 nm and 284 nm (curve a) [32]. When the Au NPs were dispersed onto the MoS_2_ nanosheets surface, a shoulder peak at 525 nm (curve b) due to the surface plasmon resonance peak of Au NPs [33, 34]. Thus, these results confirmed that the Au NPs were reduced over MoS_2_ nanosheets successfully. In addition, Au@MoS_2_ nanosheets could greatly increase the electro-active surface area and provide more active sites for biomolecules immobilization. As shown in Fig. 2D, the peak potential linearly increased with the increasing scan rate and plotted against the square root of scan rate (Fig. 2E). According to the equation of Randles-Sevcik *I*_p.a._ *=* 2.69 × 10^5^*AD*^1/2^*n*^3/2^*v*^1/2^*c* (where *I*_p.a._ is the oxidation peak current (µA), *A* is the electrochemical active area (cm^2^), *D* is the diffusion coefficient of Fe(CN)_6_^3−/4−^ (7.6 × 10^− 6^ cm^2^ s^− 1^), *n* is the transferred electron number of Fe(CN)_6_^3−/4−^ (n = 1), *v* is the scan rate (V s^− 1^), and *c* is the original concentration of Fe(CN)_6_^3−/4−^), the electrochemical active area of the modified GCE (Au@MoS_2_/GCE) was calculated as 3.17 mm^2^, which was larger than that of bare GCE (2.71 mm^2^) [35].

### Amplification mechanism of the multi-function PtCo/CdS@GO luminophores

The multi-function PtCo/CdS@GO luminophores have been in situ synthesized by co-doping with Pt and Co nanozymes simultaneously, in which PtCo was employed as an efficient peroxidase-like nanozymes toward H_2_O_2_, CdS was used as a new type of ECL luminophore, while GO was utilized as nanocarrier to load a large amount of PtCo/CdS and ensure their dispersion. Specifically, in K_2_S_2_O_8_/H_2_O_2_ coreactants system, K_2_S_2_O_8_ played a leading role (OH^•^ acceptor) in the process of promoting luminescence, while H_2_O_2_ played an auxiliary role (OH^•^ donor). The multi-function PtCo/CdS@GO luminophores (PtCo) could accelerate electrochemical redox processes of H_2_O_2_ to generate massive of transient reactive intermediates (OH^•^), and then OH^•^ rapidly reduced S_2_O_8_^2−^ to produce more strong oxidant (SO_4_^•−^) with sufficiently negative electrode potential, thus enhancing the ECL emission of PtCo/CdS@GO luminophores (CdS). Based on the synergistic effect of K_2_S_2_O_8_ and H_2_O_2_, the ECL intensity of the PtCo/CdS@GO luminophores was greatly increased. Furthermore, GO as a novel signal amplification label and nanocarrier could further amplify the ECL emission of the PtCo/CdS@GO luminophores. In addition, the amplification mechanism of the multi-function PtCo/CdS@GO luminophores in ECL immunoassay was as follows:

1 $${\text{S}}_{{\text{2}}} {{\text{O}}_{{\text{8}}}} ^{{{\text{2}} - }} + {\text{e}}^{ - } \to {{\text{SO}}_{{\text{4}}}} ^{{ \bullet - }} + {{\text{SO}}_{{\text{4}}}} ^{{{\text{2}} - }}$$

2 $${\text{H}}_{{\text{2}}} {\text{O}}_{{\text{2}}} + {\text{e}}^{ - } \to {\text{OH}}^{ \bullet } + {\text{OH}}^{ - }$$

3 $${\text{OH}}^{ \bullet } + {\text{S}}_{{\text{2}}} {{\text{O}}_{{\text{8}}}} ^{{{\text{2}} - }} \to {{\text{SO}}_{{\text{4}}}} ^{{ \bullet - }} + {{\text{HSO}}_{{\text{4}}}} ^{ - } + {\text{1}}/{\text{2O}}_{{\text{2}}}$$

4 $${{\text{SO}}_{{\text{4}}}} ^{{ \bullet - }} + {\text{PtCo}}/{\text{CdS}}@{\text{GO}} + {\text{e}}^{ - } \to {\text{PtCo}}/{\text{CdS}}@{\text{GO}}* + {{\text{SO}}_{{\text{4}}}} ^{{{\text{2}} - }}$$

5 $${\text{PtCo}}/{\text{CdS}}@{\text{GO}}* \to {\text{PtCo}}/{\text{CdS}}@{\text{GO}} + hv$$

**Fig. 3:**
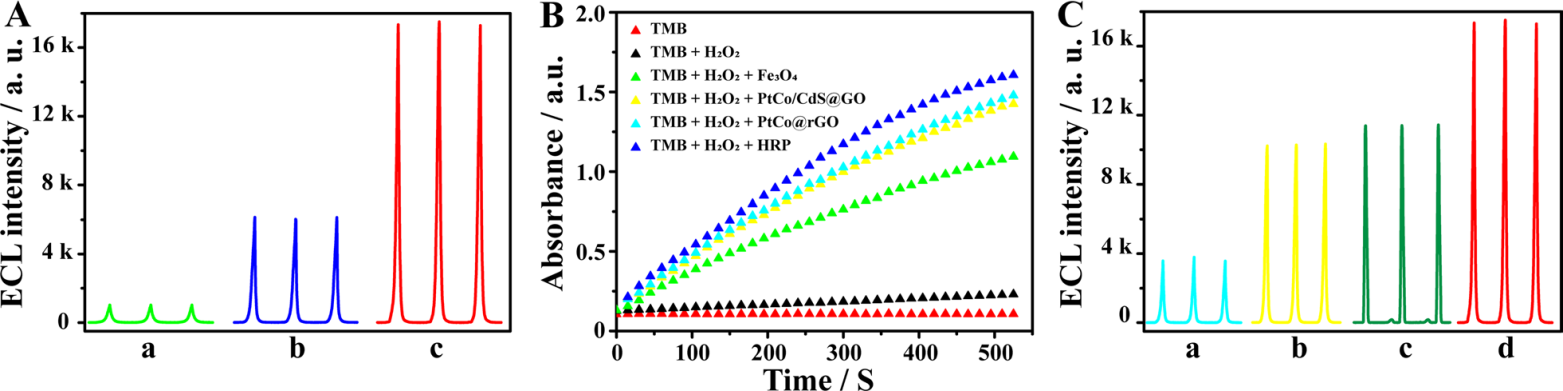
Investigation of the multi-function PtCo/CdS@GO luminophores. **A** The ECL responses of PtCo/CdS@GO luminophores in 0.01 M PBS (pH 7.4) containing 5 mM H_2_O_2_ (a), 0.1 M K_2_S_2_O_8_ (b), 5 mM H_2_O_2_ and 0.1 M K_2_S_2_O_8_ (c). **B** Time-dependent absorbance of oxTMB upon the addition of Fe_3_O_4_, PtCo/CdS@GO, PtCo/rGO and HRP in TMB/H_2_O_2_ system. **C** The ECL responses of CdS NCs (a), PtCo/CdS NCs (b), PtCo/CdS@GO (c, Cd/S = 1/4) and PtCo/CdS@GO luminophores (d, Cd/S = 1/1) in 0.01 M PBS (pH 7.4) containing 5 mM H_2_O_2_ and 0.1 M K_2_S_2_O_8_. The ECL experiments were scanned from − 1.6 V to − 0.6 V at a scan rate of 0.1 V s^− 1^

Moreover, in order to further investigate the superiority of K_2_S_2_O_8_/H_2_O_2_ coreactants, K_2_S_2_O_8_ or H_2_O_2_ as an individual coreactant had been compared in the PtCo/CdS@GO luminophores-based ECL immunosensor. As the results shown in Fig. 3A, when K_2_S_2_O_8_ or H_2_O_2_ as an individual coreactant respectively, the ECL intensity in H_2_O_2_ system (curve a, 1027 a.u) was lower than that in K_2_S_2_O_8_ (curve b, 6138 a.u). While in K_2_S_2_O_8_/H_2_O_2_ coreactants system (curve c, 17,343 a.u), the ECL intensity of PtCo/CdS@GO luminophores remarkably enhanced, resulting in ca. 2.8- and 16.9-fold enhanced compared with that of K_2_S_2_O_8_ and H_2_O_2_, respectively. In summary, these results indicated synergistic effect of K_2_S_2_O_8_ and H_2_O_2_ in PtCo/CdS@GO luminophores ECL emission.

### Comparison of the ECL responses with different nanomaterials

A typical catalytic oxidation reaction of the peroxidase substrate 3, 3′, 5, 5′-tetramethylbenzidine (TMB) by H_2_O_2_ was performed to investigate the peroxidase-like activity of Fe_3_O_4_ (classic nanozyme, Additional file 1: Figure S3A), PtCo/CdS@GO (this work), PtCo/rGO nanozyme (our previous work, Additional file 1: Figure S3B) [6] and horseradish peroxidase (HRP). As shown in Fig. 3B, when HRP was added to TMB/H_2_O_2_ system, a highest absorbance slope was obtained, which was significantly higher than that of Fe_3_O_4_ and slightly higher than that of PtCo/CdS@GO and PtCo/rGO. These results showed that the PtCo/CdS@GO possessed a comparable catalytic activity with HRP.

Moreover, in order to explore the superiority of PtCo/CdS@GO luminophores towards K_2_S_2_O_8_/H_2_O_2_ coreactants ECL system, we incubated different nanomaterials including CdS NCs [36], PtCo/CdS NCs, PtCo/CdS@GO (molar ratio: Cd/S = 1/4) and PtCo/CdS@GO luminophores (molar ratio: Cd/S = 1/1, this work) on the electrodes and compared their ECL performance. As shown in Fig. 3C, the highest ECL intensity about 17,343 a.u. was observed from the developed PtCo/CdS@GO luminophores (curve d), which was higher than that of above-mentioned nanomaterials such as CdS NCs (curve a, 3799 a.u), PtCo/CdS NCs (curve b, 10,333 a.u) and PtCo/CdS@GO (curve c, 11,257 a.u). These results were ascribed to the enhanced charge transfer and preeminent nanoparticles loading capacity of GO, which could effectively increase the luminescence efficiency of PtCo/CdS@GO. These results indicated that the multi-function PtCo/CdS@GO luminophores possessed high peroxidase-like activity and enhanced electron transfer, which could evidently enhance luminescence efficiency [27, 28]. Furthermore, GO had large specific surface area to support massive PtCo/CdS NCs and provided superior stability to ensure the dispersion of PtCo/CdS@GO luminophores in the ECL system.

### Electrochemical characterization of the ECL immunosensor

**Fig. 4:**
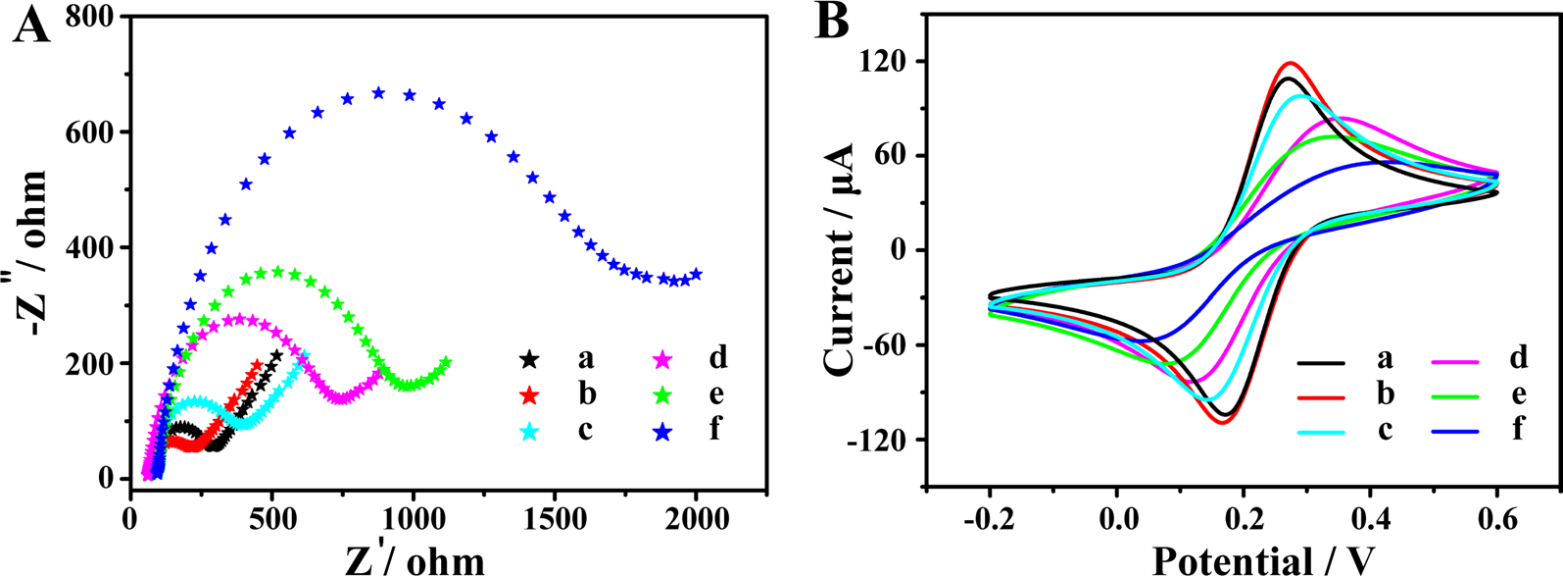
Characterization of the fabrication process of the immunosensor. **A** EIS and **B** CV curves of bare GCE (a), Au@MoS_2_/GCE (b), MPO/Au@MoS_2_/GCE (c), BSA/MPO/Au@MoS_2_/GCE (d), anti-MPO/BSA/MPO/Au@MoS_2_/GCE (e) and anti-Ab/PtCo/CdS@GO/anti-MPO/BSA/MPO/Au@MoS_2_/GCE (f) in PBS (0.01 M, pH 7.4) containing 5 mM Fe(CN)_6_^3−/4−^ and 0.1 M KCl. Z**ʹ** = real impedance component (Z_RE_); − Z**ʺ** = imaginary impedance component (Z_IM_). EIS measurements were carried out with a frequency range of 0.1–100 kHz and amplitude of 50 mV. The CV experiments were scanned from − 0.2 to 0.6 V vs Ag/AgCl reference at a scan rate of 0.1 V s^− 1^

EIS and CV were recommended as an ideal analysis tool to verify the preparation of the ECL immunosensor. As shown in Fig. 4A, EIS was performed to monitor the fabrication process of the immunosensor, and the semicircle diameter (SD) is positively correlated with the electron-transfer resistance (R_et_) at the electrode interface in Nyquist plots [37]. When Au@MoS_2_ nanosheets were immobilized onto the surface of the GCE, a smaller SD was obtained compared with the bare GCE (curve a *via* curve b) due to its excellent electroconductivity. After sequentially assembling with MPO (curve c), BSA (curve d) and target anti-MPO (curve e), the Ret successively increased due to the biomolecules impeding electron transfer. Next, when anti-Ab/PtCo/CdS@GO bioconjugates were incubated with the modified electrode, a great increase of SD was observed (curve f), demonstrating that the electron transfer was further restricted. Moreover, in order to verify the results of EIS, CV experiments was adopted to characterize the ECL immunosensor. Figure 4B showed the CV curves of different modified electrodes in the same electrolyte of EIS. And the CV results were well-consistent with that of EIS, further proving the successful constructed of the ECL immunosensor.

### Optimization of the reaction conditions

**Fig. 5:**
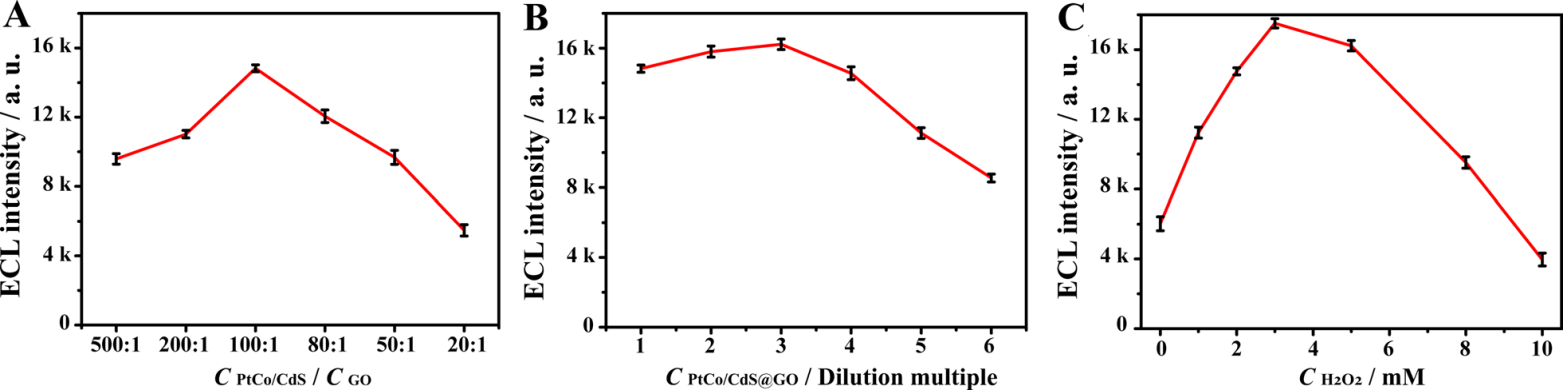
Optimization of the detection conditions. **A** The concentration proportion of PtCo/CdS NCs and GO. **B** The dilution multiple of PtCo/CdS@GO luminophores. **C** The concentration of H_2_O_2_ in K_2_S_2_O_8_/H_2_O_2_ coreactants

To explore the optimal analytical performance of the ECL immunosensor, several vital experimental parameters were systematically optimized in the whole ECL immunoassay system, including the concentration proportion of PtCo/CdS NCs and GO, the dilution ratio of PtCo/CdS@GO luminophores and the concentration of H_2_O_2_ in K_2_S_2_O_8_/H_2_O_2_ coreactants. As shown in Fig. 5A, with the concentration proportion of GO from 500:1 to 100:1, the ECL intensity gradually increased. This was because the introduction of GO enhanced the charge transfer efficiently and repressed the recombination of electron-holes pairs. However, the ECL intensity gradually decreased along with the increase of GO concentration proportion from 100:1 to 20:1, which was because the excess GO might shield the active sites of PtCo/CdS NCs surface and reduce the ECL intensity of the PtCo/CdS@GO luminophores. Thus, 100:1 of PtCo/CdS NCs and GO was chosen as the optimum proportion in this experiment. Moreover, in order to obtain better dispersion and higher ECL signal, the dilution ratio of PtCo/CdS@GO luminophores was further investigated. As displayed in Fig. 5B, a gradually increased ECL intensity with the dilution ratio of PtCo/CdS@GO luminophores, and the maximum ECL intensity was achieved at 3 times dilution (3.33 mg mL^− 1^). This was because the high concentration of PtCo/CdS@GO luminophores would form some granular precipitations after mixing with the anti-Ab, which could reduce the ECL signal. Thus, three times dilution of PtCo/CdS@GO luminophores was selected as the optimum reaction condition while presenting better stability and higher luminescence efficiency in the ECL immunoassay system. Moreover, in K_2_S_2_O_8_/H_2_O_2_ coreactants system, K_2_S_2_O_8_ played a leading role (OH^•^ acceptor) in the process of promoting luminescence, while H_2_O_2_ played an auxiliary role (OH^•^ donor) [38]. Figure 5C illustrated the effect of the concentration of H_2_O_2_ in K_2_S_2_O_8_/H_2_O_2_ coreactants on the analytical performance of the ECL immunosensor. When the H_2_O_2_ concentration increased from 0 to 3 mM, the ECL intensity gradually increased, and then reduced rapidly as the H_2_O_2_ concentration continued to increase. This was because the excessive H_2_O_2_ produced large amounts of OH^•^, which in turn could scavenge SO_4_^•−^ (eq OH^•^ + SO_4_^•−^ → HSO_4_^−^ + 1/2O_2_), resulting in a decrease in the ECL intensity. Additionally, above-mentioned results were also supported by some similar literatures[38, 39]. Therefore, 3 mM of H_2_O_2_ in K_2_S_2_O_8_/H_2_O_2_ coreactants was chosen as the optimal concentration.

### Analytical performance of the ECL immunosensor

**Fig. 6:**
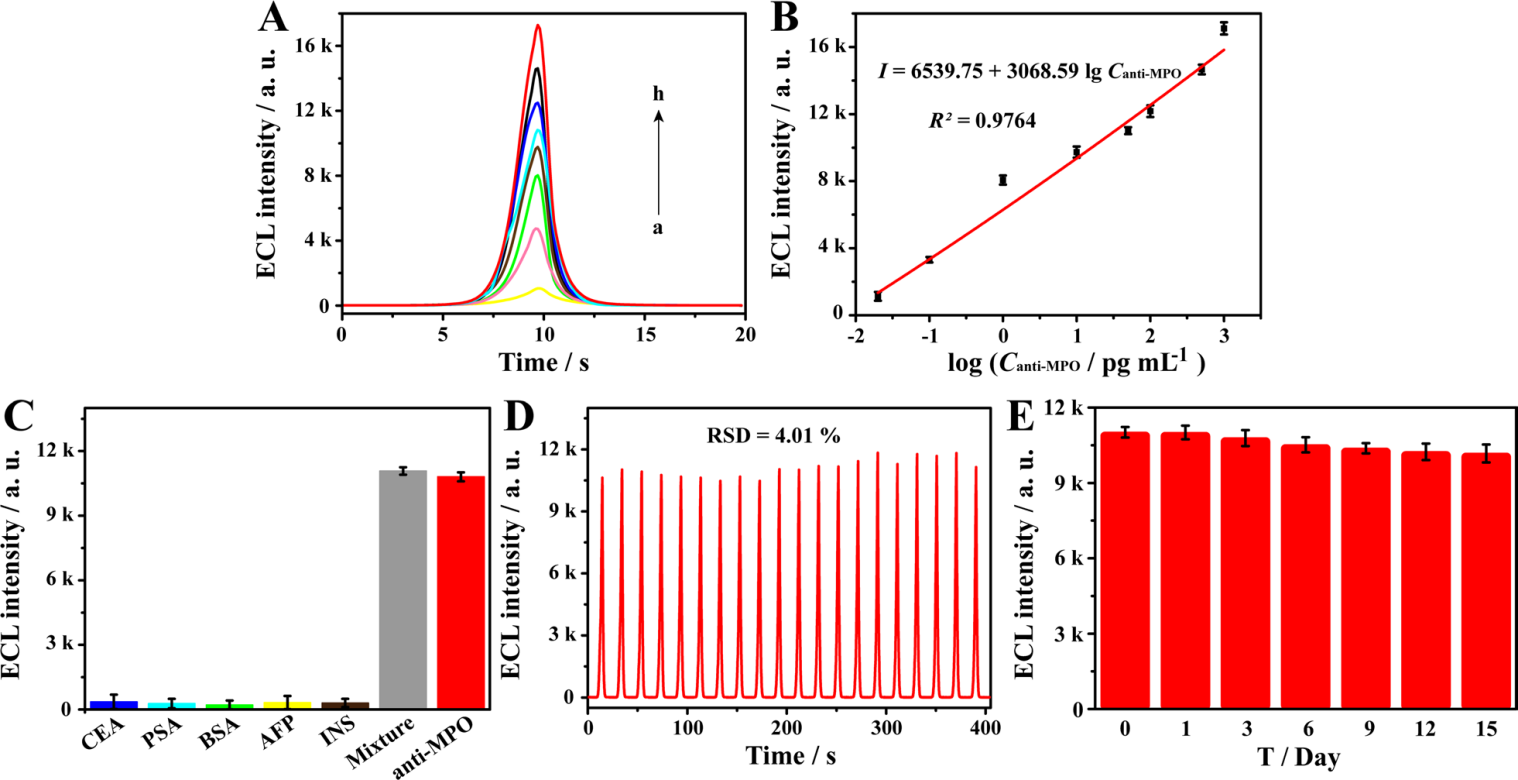
Evaluation of analytical performance of the ECL immunosensor. **A** ECL intensity of the proposed immunosensor with 0.02, 0.1, 1.0, 10, 50, 100, 500 and 1000 pg mL^− 1^ of anti-MPO. **B** Calibration plot of the ECL intensities vs. the log C_anti−MPO_. **C** The cross-reactivity of the immunosensor against different interfering proteins (50 pg mL^− 1^ CEA, 50 pg mL^− 1^ PSA, 50 pg mL^− 1^ BSA, 50 pg mL^− 1^ AFP and 50 pg mL^− 1^ INS) and their mixtures (50 pg mL^− 1^ CEA, 50 pg mL^− 1^ PSA, 50 pg mL^− 1^ BSA, 50 pg mL^− 1^ AFP, 50 pg mL^− 1^ INS and 50 pg mL^− 1^ anti-MPO). **D** Investigation of the stability of the immunosensor by detecting 50 pg mL^− 1^ anti-MPO with 20 consecutive cycles. Data were expressed as mean ± standard deviations, sample replicates n = 3. **E** Long-term stability of the ECL immunosensor for anti-MPO detection in 4 mL 0.01 M PBS (pH 7.4) containing 3 mM H_2_O_2_ and 0.1 M K_2_S_2_O_8_ with a potential from − 1.6 V to -0.6 V at a scan rate of 0.1 V s^− 1^

Under the optimal experimental conditions, different concentrations of anti-MPO in solution were detected by the proposed ECL immunosensor for the purpose of quantitative analysis. Figure 6A showed that the ECL intensities progressively increased with the increase of anti-MPO concentrations covering the range of 0.02 to 1000 pg mL^− 1^, and displayed an excellent linear relationship between the ECL intensity change and the function of log C_anti−MPO_ (Fig. 6B). The fitted linear regression equation was *I* = 6539.75 + 3068.59 log C_anti−MPO_ (*R*^*2*^ = 0.9764), where *I* was ECL intensity and log C_anti−MPO_ was the logarithm of anti-MPO concentrations. The limit of detection (LOD) was 7.39 fg mL^− 1^ calculated as the signal to noise ratio (S/N) of 3. More importantly, a comparison of ECL immunosensors in different amplification strategies had been performed to verify the analytical performance of the developed ECL immunosensors. As shown Additional file 1: Table S1, the developed ECL immunosensor exhibits a wider linear range (20 fg mL^− 1^ − 1 ng mL^− 1^) and lower limit of detection (7.39 fg mL^− 1^). This was mainly attributed to the multi-function PtCo/CdS@GO luminophores with superior stability in aqueous solutions, efficient catalytic capacity to H_2_O_2_ and the amplified ECL emission by the nanocarrier GO.

The specificity of the proposed ECL immunosensor was investigated against carcinoembryonic antigen (CEA), prostate specific antigen (PSA), BSA, alpha-fetoprotein (AFP), insulin (INS) and their mixtures containing anti-MPO. Different interfering proteins and 50 pg mL^− 1^ anti-MPO were incubated with the proposed ECL immunosensor, respectively. As shown in Fig. 6C, anti-MPO displayed an obvious ECL response compared to the interfering proteins, indicating that this established ECL immunosensor demonstrated satisfactory specificity for anti-MPO detection. In addition, the stability as another key parameter of the ECL immunosensor was evaluated under 20 consecutive cycles with 50 nM anti-MPO. Meanwhile, as shown in Fig. 6D, the relative standard deviation (RSD) value was as low as 4.01 %, displaying an acceptable stability, demonstrating that the multi-function PtCo/CdS@GO luminophores could maintain high-efficiency luminescence performance in the ECL immunosensor.

Furthermore, the long-term stability of the ECL immunosensor was also investigated by storing BSA/MPO/Au@MoS_2_/GCE at 4 ºC in different time intervals (0, 1, 3, 6, 9, 12 and 15 days), and then incubated with target (anti-MPO, 50 pg mL^− 1^) and anti-Ab/PtCo/CdS@GO. As the results illustrated in Fig. 6E, the ECL intensity of the fabricated immunosensor was not significantly reduced, and remained at 92.37 % of its initial ECL intensity after 15 days, indicating that the ECL immunosensor had an acceptable long-term stability.

### Application in clinical serum samples

To further estimate the practicability of the proposed ECL immunosensor in a complex biological matrix, recovery experiments were executed by successively adding anti-MPO in clinical serum samples with a known gradient concentration. As shown in Table 1, the concentrations of anti-MPO were accurately quantified by the fitted linear equation in Fig. 6B (*I* = 6539.75 + 3068.59 log C_anti−MPO_), and the recoveries were obtained from 95.54 to 106.37 % with the RSD of 0.84 to 3.20 % (n = 3). These results demonstrated that the proposed ECL immunosensor could serve as an alternative method for ultrasensitive detection of anti-MPO held a great potential in clinical application.

**Table 1:** Determination of anti-MPO with different concentrations in normal serum samples by the proposed ECL inmmunosensor

Sample	Standard solution (fg mL^− 1^)	ECL intensity (a.u.)	Calculated (fg mL^− 1^)	Recovery (%)	RSD (%)
1	50.0	2486.7	47.77	95.54	0.84
2	500.0	5631.3	505.78	101.16	3.20
3	5.00 × 10^3^	8727.0	5.16 × 10^3^	103.23	2.74
4	7.50 × 10^4^	12260.3	7.32 × 10^4^	97.54	2.31
5	2.00 × 10^5^	13,683	2.13 × 10^5^	106.37	0.92

## Conclusions

In this work, an ultrasensitive ECL immunosensor based on the dual amplification strategies of multi-function PtCo/CdS@GO luminophores and K_2_S_2_O_8_/H_2_O_2_ coreactants has been developed for anti-MPO detection. Benefiting from several considerable advantages of PtCo/CdS@GO luminophores, including extraordinary peroxidase-like activity toward K_2_S_2_O_8_/H_2_O_2_, high-efficiency luminescence performance and superior stability in aqueous solutions, the ECL immunosensor for anti-MPO detection displayed excellent analytical performance with linear concentration range from 0.02 to 1000 pg mL^− 1^ and LOD down to 7.39 fg mL^− 1^. More importantly, the introduction of multi-function PtCo/CdS@GO luminophores not only offers a successful application for anti-MPO detection in clinical serum samples, but also provides a new concept to design other semiconductor nanomaterials in biomarker detection systems (e.g., ECL immunoassay, photoelectrochemical immunoassay), drug delivery systems and disease treatment systems. However, there are still some challenges in the future, including simplifying the synthesis steps of PtCo/CdS@GO luminophores and the influence of different molar ratio of Pt/Co on peroxidase-like activity and ECL emission. In conclusion, the ECL immunosensor hold great potential for biomarkers detection in clinical application.

## Supplementary Information

**Additional file 1.** Characterization of MoS_2_ and Au@MoS_2_ nanosheets.

## Data Availability

The datasets used and/or analysed during the current study are available from the corresponding author on reasonable request.

## References

[1] Nakazawa D, Masuda S, Tomaru U, Ishizu A (2018). Pathogenesis and therapeutic interventions for ANCA-associated vasculities. Nat Rev Rheumatol.

[2] Guillevin L, Pagnoux C, Karras A, Khouatra C, AumaiTre O, Cohen P, Maurier F, Ravaud P, Mouthon L (2014). Rituximab versus azathioprine for maintenance in ANCA-associated vasculitis. N Engl J Med.

[3] Moiseev S, Bossuyt X, Arimura Y, Blockmans D, Csernok E, Damoiseaux J, Emmi G, Flores-Suárez L, Hellmich B, Jayne D, Jennette J, Little M, Mohammad A, Moosig F, Novikov P, Pagnoux C, Radice A, Sada K, Segelmark M, Shoenfeld Y, Sinico R, Specks U, Terrier B, Tzioufas A, Vaglio A, Zhao MJ, Cohen Tervaert J (2020). International consensus on ANCA Testing in eosinophilic granulomatosis with polyangiitis. Am J Respir Crit Care Med.

[4] Bossuyt X, Cohen Tervaert JW, Arimura Y, Blockmans D, Flores-Suarez LF, Guillevin L, Hellmich B, Jayne D, Jennette JC, Kallenberg CGM, Moiseev S, Novikov P, Radice A, Savige JA, Sinico RA, Specks U, van Paassen P, Zhao MH, Rasmussen N, Damoiseaux J, Csernok E (2017). Position paper: revised 2017 international consensus on testing of ANCAs in granulomatosis with polyangiitis and microscopic polyangiitis. Nat Rev Rheumatol.

[5] Savige J, Dimech W, Fritzler M, Goeken J, Hagen EC, Jennette JC, McEvoy R, Pusey C, Pollock W, Trevisin M, Wiik A, Wong R (2003). Addendum to the International consensus statement on testing and reporting of antineutrophil cytoplasmic antibodies. Am J Clin Pathol.

[6] Yang W, Peng Q, Guo Z, Wu H, Ding S, Chen Y, Zhao M (2019). PtCo nanocubes/reduced graphene oxide hybrids and hybridization chain reaction-based dual amplified electrochemiluminescence immunosensing of antimyeloperoxidase, Biosens. Bioelectron.

[7] Farka Z, Juřík T, Kovář D, Trnková L, Skládal P (2017). Nanoparticle-based immunochemical biosensors and assays: recent advances and challenges. Chem Rev.

[8] Li L, Chen Y, Zhu JJ (2016). Recent advances in electrochemiluminescence analysis. Anal Chem.

[9] Gu W, Wang H, Jiao L, Wu Y, Chen Y, Hu L, Gong J, Du D, Zhu C (2020). Single-atom iron boosts electrochemiluminescence. Angew Chem Int Ed Engl.

[10] Irkham A, Fiorani G, Valenti N, Kamoshida F, Paolucci Y, Einaga (2020). Electrogenerated chemiluminescence by in situ production of coreactant hydrogen peroxide in carbonate aqueous solution at a boron-doped diamond electrode. J Am Chem Soc.

[11] Elshaari A, Zadeh I, Fognini A, Reimer M, Dalacu D, Poole P, Zwiller V, Jöns K (2017). On-chip single photon filtering and multiplexing in hybrid quantum photonic circuits. Nat Commun.

[12] Kim Y, Kim H, Cho Y, Ryoo J, Park C, Kim P, Kim Y, Lee S, Li Y, Park S, Yoo Y, Yoon D, Dorgan V, Pop E, Heinz T, Hone J, Chun S, Cheong H, Lee S, Bae M, Park Y (2015). Bright visible light emission from graphene. Nat Nanotechnol.

[13] Huang Z, Li X, Mahboub M, Hanson K, Nichols V, Le H, Tang M, Bardeen C (2015). Hybrid molecule-nanocrystal photon upconversion across the visible and near-infrared. Nano Lett.

[14] Low J, Dai B, Tong T, Jiang C, Yu J (2019). In situ irradiated X-ray photoelectron spectroscopy investigation on a direct Z-scheme TiO /CdS composite film photocatalyst. Adv Mater.

[15] He T, Qiu X, Li J, Pang G, Wu Z, Cheng J, Zhou Z, Hao J, Liu H, Ni Y, Li L, Lin X, Hu W, Wang K, Chen R (2019). Water-soluble chiral CdSe/CdS dot/rod nanocrystals for two-photon fluorescence lifetime imaging and photodynamic therapy. Nanoscale.

[16] Feng J, Li F, Li X, Wang Y, Fan D, Du B, Li Y, Wei Q (2018). Label-free photoelectrochemical immunosensor for NT-proBNP detection based on La-CdS/3D ZnInS/Au@ZnO sensitization structure, Biosens. Bioelectron.

[17] Arfin H, Kaur J, Sheikh T, Chakraborty S, Nag A (2020). Bi^3+^ -Er^3+^ and Bi^3+^ -Yb^3+^ codoped Cs AgInCl double perovskite near-infrared emitters. Angew Chem Int Ed Engl.

[18] Zhang J, Yuan X, Si M, Jiang L, Yu H (2020). Core-shell structured cadmium sulfide nanocomposites for solar energy utilization. Adv Colloid Interface Sci.

[19] Hanifi D, Bronstein N, Koscher B, Nett Z, Swabeck J, Takano K, Schwartzberg A, Maserati L, Vandewal K, van de Burgt Y, Salleo A, Alivisatos A (2019). Redefining near-unity luminescence in quantum dots with photothermal threshold quantum yield. Science.

[20] Dai H, Ma Z, Wang F, Zhong Y, Salazar F, Li J, Zhang M, Ren F, Wu A (2020). Advancing nanomedicine with cross-link functionalized nanoparticles for rapid excretion. Angew Chem Int Ed Engl.

[21] Gao L, Zhuang J, Nie L, Zhang J, Zhang Y, Gu N, Wang T, Feng J, Yang D, Perrett S, Yan X (2007). Intrinsic peroxidase-like activity of ferromagnetic nanoparticles. Nat Nanotechnol.

[22] Wu J, Wang X, Wang Q, Lou Z, Li S, Zhu Y, Qin L, Wei H (2019). Nanomaterials with enzyme-like characteristics (nanozymes): next-generation artificial enzymes (II). Chem Soc Rev.

[23] Wei H, Wang E (2013). Nanomaterials with enzyme-like characteristics (nanozymes): next-generation artificial enzymes. Chem Soc Rev.

[24] Wang H, Wan K, Shi X (2019). Recent advances in nanozyme research. Adv Mater.

[25] Zhou X, Fang Y, Cai X, Zhang S, Yang S, Wang H, Zhong X, Fang Y (2020). In situ photodeposited construction of Pt–CdS/g-C3N4–MnOx composite photocatalyst for efficient visible-light-driven overall water splitting. ACS Appl Mater Interfaces.

[26] Qin Y, Zhang X, Dai X, Sun H, Yang Y, Li X, Shi Q, Gao D, Wang H, Yu NF, Sun SG (2016). Graphene oxide-assisted synthesis of Pt-Co alloy nanocrystals with high-index facets and enhanced electrocatalytic properties. Small.

[27] Jia Q, Zhao Z, Cao L, Li J, Ghoshal S, Davies V, Stavitski E, Attenkofer K, Liu Z, Li M, Duan X, Mukerjee S, Mueller T, Huang Y (2018). Roles of Mo surface dopants in enhancing the ORR performance of octahedral PtNi nanoparticles. Nano Lett.

[28] Yoo TY, Yoo JM, Sinha AK, Bootharaju MS, Jung E, Lee HS, Lee BH, Kim J, Antink WH, Kim YM, Lee J, Lee E, Lee DW, Cho SP, Yoo SJ, Sung YE, Hyeon T (2020). Direct synthesis of intermetallic platinum-alloy nanoparticles highly loaded on carbon supports for efficient electrocatalysis. J Am Chem Soc.

[29] Liu Q, Shi J, Sun J, Wang T, Zeng L, Jiang G (2011). Graphene and graphene oxide sheets supported on silica as versatile and high-performance adsorbents for solid-phase extraction. Angew Chem Int Ed Engl.

[30] Shi L, Mu C, Gao T, Chai W, Sheng A, Chen T, Yang J, Zhu X, Li G (2019). Rhodopsin-Like ionic gate fabricated with graphene oxide and isomeric DNA switch for efficient photocontrol of ion transport. J Am Chem Soc.

[31] Kang KT, Park J, Suh D, Choi WS (2019). Synergetic behavior in 2D layered material/complex oxide heterostructures. Adv Mater.

[32] Liu T, Wang C, Gu X, Gong H, Cheng L, Shi X, Feng L, Sun B, Liu Z (2014). Drug delivery with PEGylated MoS_2_ nano-sheets for combined photothermal and chemotherapy of cancer. Adv Mater.

[33] Su S, Zhang C, Yuwen L, Chao J, Zuo X, Liu X, Song C, Fan C, Wang L (2014). Creating SERS hot spots on MoS(2) nanosheets with in situ grown gold nanoparticles. Appl Mater Interfaces.

[34] Hindson CM, Chevillet JR, Briggs HA, Gallichotte EN, Ruf IK, Hindson BJ, Vessella RL, Tewari M (2013). Absolute quantification by droplet digital PCR versus analog real-time PCR. Nat Methods.

[35] Li X, Shen J, Wu C, Wu K (2019). Ball-mill-exfoliated graphene: tunable electrochemistry and phenol sensing. Small.

[36] Zhang YY, Zhou H, Wu P, Zhang HR, Xu JJ, Chen HY (2014). In situ activation of CdS electrochemiluminescence film and its application in H_2_S detection. Anal Chem.

[37] Chen J, Zhang J, Yang H, Fu F, Chen G (2010). A strategy for development of electrochemical DNA biosensor based on site-specific DNA cleavage of restriction endonuclease. Biosens Bioelectron.

[38] Nie Y, Liu Y, Zhang Q, Su X, Ma Q (2019). Novel coreactant modifier-based amplified electrochemiluminescence sensing method for point-of-care diagnostics of galactose. Biosens Bioelectron.

[39] Dai PP, Yu T, Shi HW, Xu JJ, Chen HY (2015). General strategy for enhancing electrochemiluminescence of semiconductor nanocrystals by hydrogen peroxide and potassium persulfate as dual coreactants. Anal Chem.

