# Supplementary material for: Multi-function PtCo nanozymes/CdS nanocrystals@graphene oxide luminophores and K2S2O8/H2O2 coreactants-based dual amplified electrochemiluminescence immunosensor for ultrasensitive detection of anti-myeloperoxidase antibody
Source: J Nanobiotechnology. 2021 Jul 29;19:225. doi: 10.1186/s12951-021-00968-4 (PMC8323290; doi:10.1186/s12951-021-00968-4)
Supplement: Supplementary file 1 — Additional file 1. Characterization of MoS2 and Au@MoS2 nanosheets. [file 12951_2021_968_MOESM1_ESM.doc]

Additional information

Multi-function PtCo nanozymes/CdS nanocrystals@graphene oxide luminophores and K_2_S_2_O_8_/H_2_O_2_ coreactants-based dual amplified electrochemiluminescence immunosensor for ultrasensitive detection of anti-myeloperoxidase antibody

Wei Yang^1,3†^, Zheng Zhou^2,3†^, Haiping Wu^3^, Changjin Liu^3^, Bo Shen^3^, Shijia Ding^3^,*, Yonglie Zhou1,[[1]](#footnote-2)*

^1^ *Department of Clinical Laboratory, Zhejiang Provincial People’s Hospital, People’s Hospital of Hangzhou Medical College, Hangzhou, 310014, China*

^2^ *Department of Clinical Laboratory, Chongqing University Three Gorges Hospital, Chongqing, 404000, China*

^3^ *Key Laboratory of Clinical Laboratory Diagnostics (Ministry of Education),* *College of Laboratory Medicine, Chongqing Medical University, Chongqing, 400016, China*

**Preparation of Au@MoS_2_ nanosheets**

The utilized Au@MoS_2_ nanosheets were synthesized according to our previous work with some modification [1]. 10 mL MoS_2_ (20 μg/mL), 120 μL sodium citrate (100 mM), 20 μL Tween-80 (100 mM) and 100 μL HAuCl_4_ (28.5 mM) solutions were mixed together and stirred vigorously. Next, the obtained mixture was heated to 60 °C for 5 min and then naturally cooled down to room temperature. Finally, the product of Au@MoS_2_ nanosheets was centrifuged (7000 rpm, 20 min) to remove excess reagents. After that, the supernatant was discarded and the precipitates (Au@MoS_2_ nanosheets) were dispersed in 5 mL deionized water.

**Preparation of the Modified GCE**

The modified GCE was fabricated as follow: firstly, the bare glassy carbon electrode (GCE, 3 mm in diameter) was polished with 0.3 μm Al_2_O_3_ slurries on a polished cloth to obtain a mirror-like surface, and then ultrasonically washed with deionized water and ethanol successively at RT for 10 min. Subsequently, the GCE was dried under high-purity nitrogen flush at RT for 30 s. After that, 10 μL Au@MoS_2_ nanosheets solution was dropped onto the cleaned GCE to form Au@MoS_2_ film when the solvent evaporated naturally at 37 °C. Then, 10 μL antigen MPO (200 μg/mL) dispersion was added onto the obtained electrode (Au@MoS_2_/GCE) and incubated overnight at 4 °C. Subsequently, in order to eliminate nonspecific binding sites of the modified electrode, 10 μL BSA blocking buffer (1.0 wt%) was dropped onto the MPO/Au@MoS_2_/GCE and incubated at RT for 1 h to eliminate nonspecific binding sites of the modified electrode. Ultimately, the modified electrode (BSA/MPO/Au@MoS_2_/GCE) was gently rinsed with 0.01 M PBS (pH 7.4) for further use.


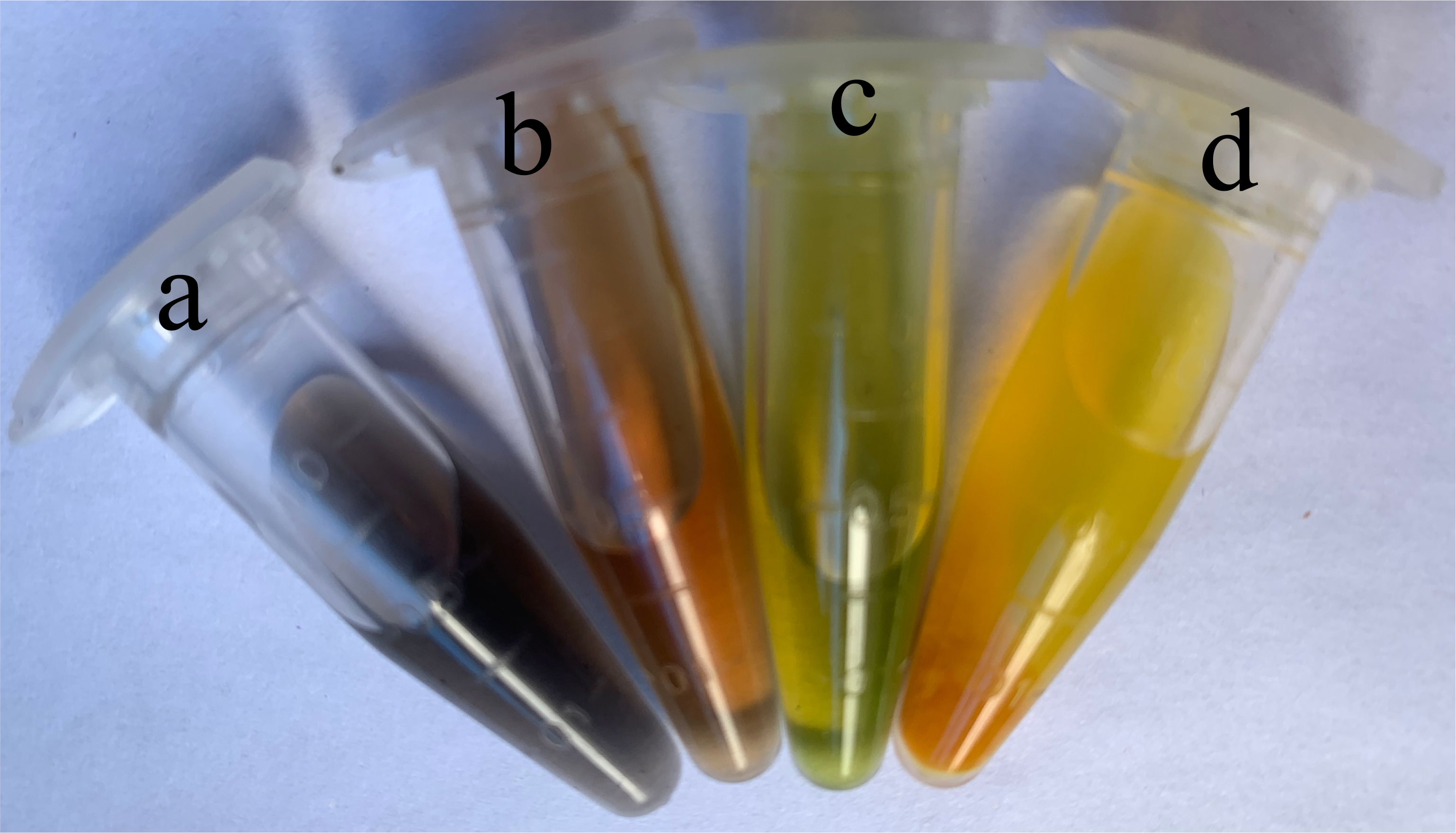


**Fig. S1** Stability characterization of PtCo NCs (a), GO (b), PtCo/CdS@GO luminophores (c) and CdS NCs (d) in aqueous solutions for 60 min.


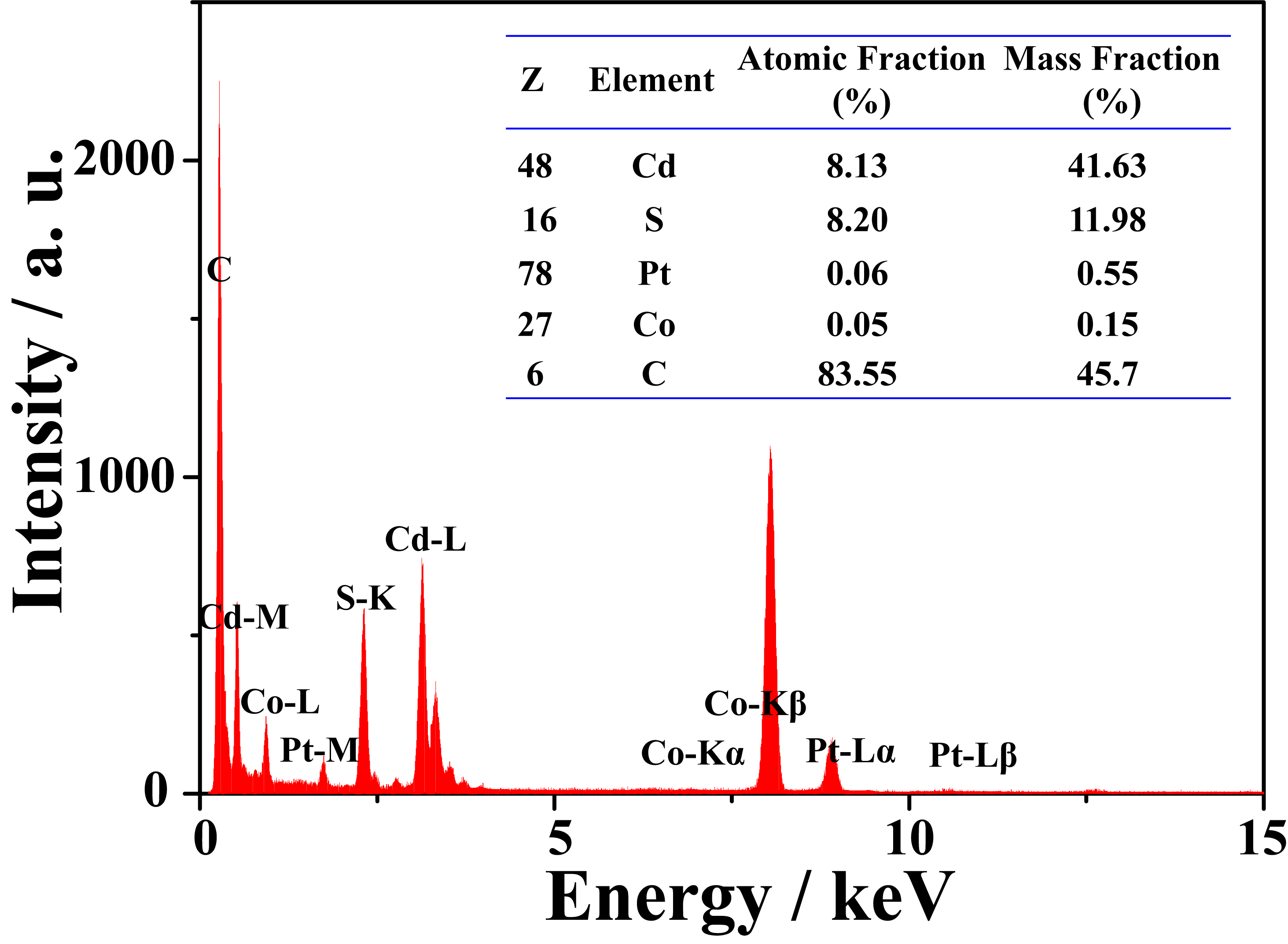


**Fig. S2** EDS elemental analysis of multi-function PtCo/CdS@GO luminophores.


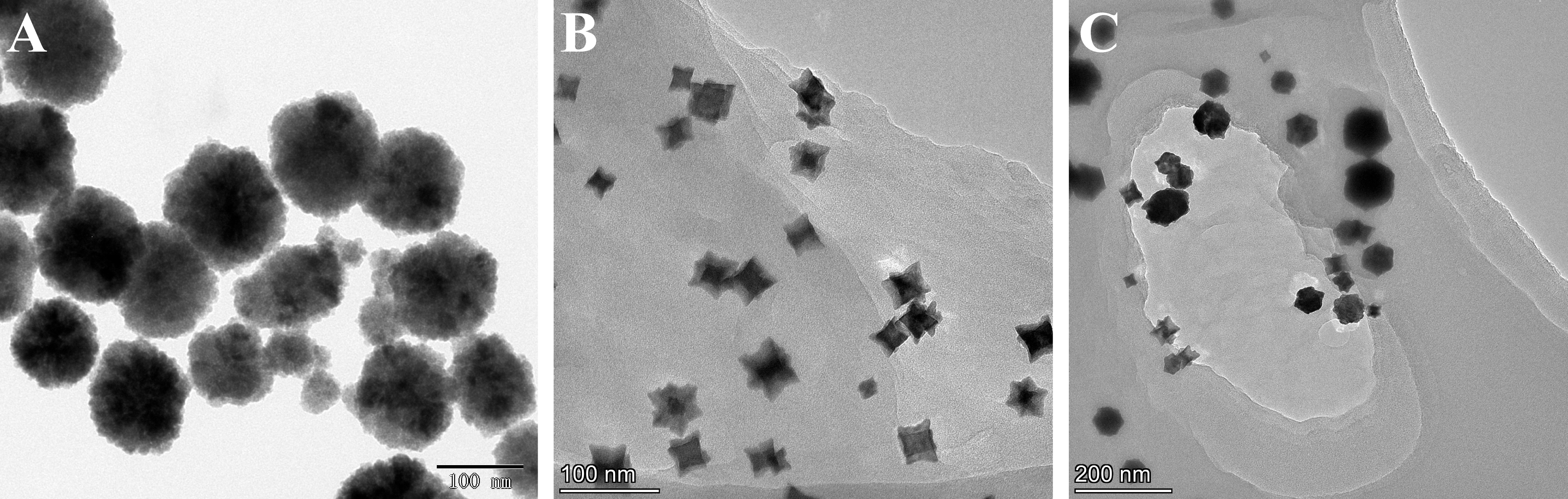


**Fig. S3** TEM images of Fe_3_O_4_ (A) and PtCo/rGO (B). Scale bar is 100 nm. (C) TEM image of PtCo/CdS@GO (Cd:S = 1/4). Scale bar is 200 nm.

**Table S1 Comparison of similar technologies (ECL immunosensors) in amplification strategy and analytical performance.**

| **Technology** | **Amplification strategy** | **Linear range** | **LOD** | **Ref.** |
| --- | --- | --- | --- | --- |
| ECL  immunosensor | CdTe and CdSe nanocrystals | 1×10^4^ fg mL^-1^ - 10 ng mL^-1^ | 1×10^3^ fg mL^-1^ | [2] |
| ECL  immunosensor | CdS-Au nanorods | 1×10^6^ fg mL^-1^ - 12 ng mL^-1^ | 6×10^5^ fg mL^-1^ | [3] |
| ECL  immunosensor | CdS/chitosan/  g-C3N4 | 1×10^3^ fg mL^-1^ - 100 ng mL^-1^ | 1.4×10^2^ fg mL^-1^ | [4] |
| ECL  immunosensor | PtCo@rGO | 50 fg mL^-1^ - 1 ng mL^-1^ | 15.68 fg mL^-1^ | Our previous work [1] |
| ECL  immunosensor | PtCo/CdS@GO | 20 fg mL^-1^ - 1 ng mL^-1^ | 7.39 fg mL^-1^ | This work |

ECL: electrochemiluminescence; LOD: limit of detection; CdTe: cadmium telluride; CdSe: cadmium selenide; CdS: cadmium sulfide; rGO: reduced graphene oxide; GO: graphene oxide.

**References**

[1] W. Yang, Q. Peng, Z. Guo, H. Wu, S. Ding, Y. Chen, M. Zhao, PtCo nanocubes/reduced graphene oxide hybrids and hybridization chain reaction-based dual amplified electrochemiluminescence immunosensing of antimyeloperoxidase, Biosens. Bioelectron. 2019; 142:111548.

[2] G. Zou, X. Tan, X. Long, Y. He, W. Miao, Spectrum-Resolved Dual-Color Electrochemiluminescence Immunoassay for Simultaneous Detection of Two Targets with Nanocrystals as Tags, Anal. Chem. 2017; 89:13024-13029.

[3] X.L. Huo, H. Yang, M.X. Li, W. Zhao, J.J. Xu, Y. Wang, X.L. Luo, H.Y. Chen, Multi-segmented CdS-Au nanorods for electrochemiluminescence bioanalysis, Nanoscale 2018; 10:19224-19230.

[4] B. Huang, X.P. Liu, J.S. Chen, C.J. Mao, H.L. Niu, B.K. Jin, Electrochemiluminescence immunoassay for the prostate-specific antigen by using a CdS/chitosan/g-C3N4 nanocomposite, Mikrochim. Acta. 2020; 187:155.

1. ^^*Correspondence: E-mail address: zhouyonglievip@163.com; dingshijia@163.com

   ^†^ Wei Yang and Zheng Zhou contributed equally to this manuscript [↑](#footnote-ref-2)
